# 1340. Yield of Repeat Blood Cultures beyond 48 Hours after Negative Initial Cultures in Patients Hospitalized on a Pediatric Hematology/Oncology Unit

**DOI:** 10.1093/ofid/ofac492.1169

**Published:** 2022-12-15

**Authors:** Cassandra S Prather, Muayad Alali

**Affiliations:** Indiana University School of Medicine, Indianapolis, Indiana; Indiana university, Carmel, Indiana

## Abstract

**Background:**

Repeat blood cultures (BCxs) beyond 48 hours are often obtained despite negative initial BCxs in hospitalized pediatric hematology/oncology patients. This study seeks to determine the yield of repeat BCxs after negative initial cultures in these patients and to characterize new positive BCxs beyond 48 hours and the clinical contexts in which they were obtained.

**Methods:**

A retrospective review utilizing MedMined Inc. Data Mining Surveillance database was conducted on all BCxs obtained on hospitalized patients on the pediatric hematology/oncology unit at Riley Hospital for Children in Indianapolis, IN from January 2015 to February 2021. Exclusion criteria are shown in Fig. 1. Patient episodes in which a new pathogen (or commensal treated by the primary team as a pathogen) was identified on a repeat BCx more than 48 hours after negative initial BCxs were further investigated via electronic medical record review.

**Figure:**
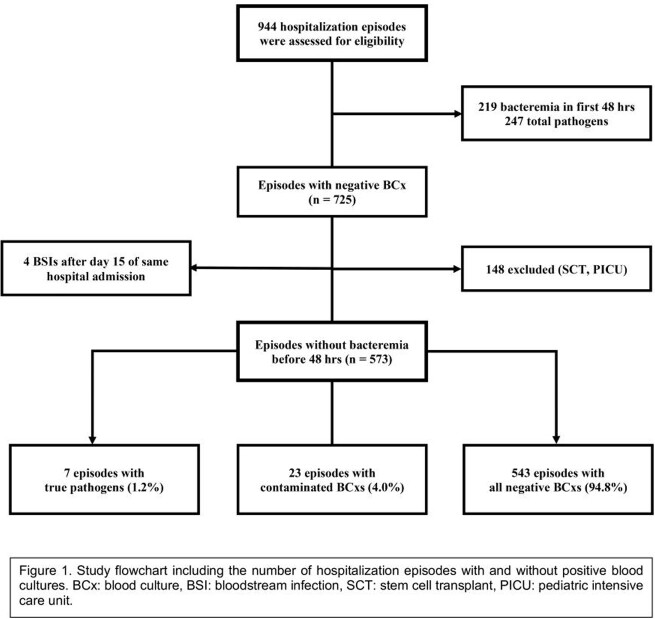

**Results:**

A total of 1,362 BCx sets were obtained beyond 48 hours in 792 patient hospitalizations, resulting in 303 positive BCxs (Fig. 2). Of these positive cultures, 193 were the same pathogen cultured on day 0 and 74 were contaminant cultures (in 4.0% (23/573) of patient hospitalizations without a positive BCx before 48 hours). Only 36 (2.6%) of positive BCxs beyond 48 hours were determined to be new pathogens, or commensals treated as pathogens, that were not cultured before 48 hours, corresponding to seven patient hospitalizations (1.2% (7/573) of patient hospitalizations without a positive BCx before 48 hours). The majority (6/7) of these patients were neutropenic and on broad spectrum antibiotics when the new positive BCxs were obtained. Fever pattern was prolonged in one patient and recurrent in six. No deaths occurred in these seven patients. All patients with new, true pathogens on BCxs beyond 48 hours (n=5) were either hemodynamically unstable (n=3) or had clinical changes (n=2, mucositis, diarrhea) the day the new positive BCx was drawn.

**Figure:**
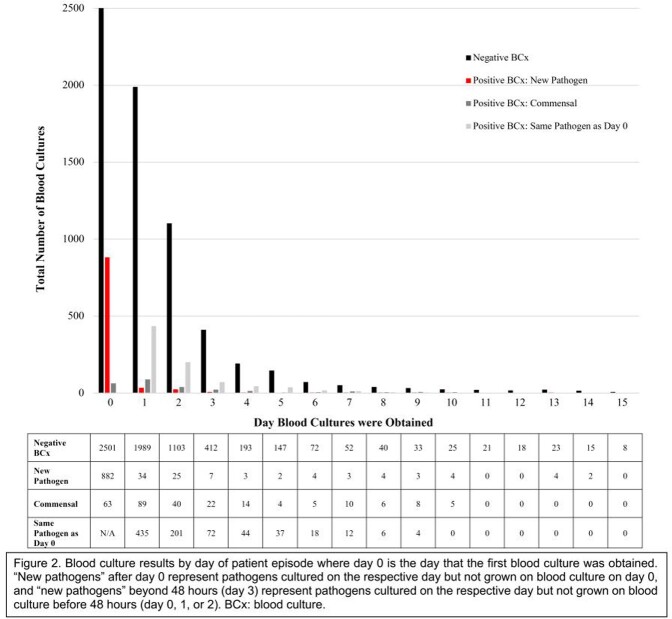

**Conclusion:**

The yield of repeat BCxs beyond 48 hours in hospitalized pediatric hematology/oncology patients with negative initial BCxs is low, while the associated costs are high. Repeat BCxs beyond 48 hours after negative initial cultures need not be obtained in febrile patients that remain hemodynamically stable and without clinical changes.

**Disclosures:**

**Cassandra S. Prather, MD**, Eli Lilly and Company: Spouse is employed by Eli Lilly and Company. Spouse owns stock in the company.|Eli Lilly and Company: Stocks/Bonds.

